# Principles of Human Physiology, with Their Application to Pathology, Hygiene and Forensic Medicine

**Published:** 1843-09-30

**Authors:** 


### BIBLIOGRAPHICAL NOTICES.

*Principles of Human Physiology, with their Applications to Pathology, Hygiéne, and Forensic Medicine. Especially designed for the use of Students. With over One Hundred Illustrations.* By William B. Carpenter, M. D., Lecturer on Physiology in the Bristol Medical School, &c. *First American Edition, with Additions by the Author, and Notes and Additions*, by Meredith Clymer, M. D., Lecturer on the Institutes of Medicine, Physician to the Philadelphia Hospital, Fellow of the College of Physicians, &c. Philadelphia: Lea & Blanchard. 1843. 8vo. pp. 618.

Within the last ten years the whole aspect of the Science of Life has changed. The revival of the application of the Microscope, to the investigation of the solid and fluid constituents of the animal fabric, and the progress of Organic Chemistry, have contributed largely to this revolution. An improved apparatus, and an advanced condition of physical science, guided by reason and reflection have enabled the Microscope to work out wonders in minute organization, and to reveal both structural and functional facts of the highest value. Restricted within proper limits, and confined to those who by organization and training are skilful manipulators, it will, as long as it is guided by mind, continue still to enlarge our knowledge on essential points, which without its aid would long remain in obscurity. To the prompt and determined march of Organic Chemistry no small share of the recent successes of Physiology are due; not that we think that the laws of life are the laws of Chemistry; far from it; for we believe that no elucidation of the truly vital processes of the economy will ever be afforded by Chemistry; but light, however, may be thrown by it on many of the organic phenomena, and we look upon it as a powerful adjuvant to Physiology, so long as it is contented to remain her handmaid, and aid her by its searching agency. An unphilosophical pretension on the part of some of the Organic Chemists has alarmed many sound physiologists; but enthusiasm or egotism on their part is no excuse for the indulgence of a pettish humour, which totally rejects their aid. We cannot do without them; and if they are arrogant and threatening, we must receive just so much from them as we want and believe to be to our purpose, and unscrupulously reject the dross. The writings of Liebig, praiseworthy and excellent in their *ensemble*, are notoriously deficient and crude in their details, and that morbid spirit for novelty so baneful to our science, gave much that was erroneous and objectionable a temporary circulation and notoriety entirely incommensurate with its merits. Still the speculations of the Professor of Giessen were useful, independent of their intrinsic value, which was often considerable, from their suggestive character, opening up new, interesting and important paths of inquiry.

This rapid advance of Physiology necessarily required new treatises, representing the actual condition of the science, and the work whose title heads this article is an endeavour to supply this want. The invaluable work of Müller, though presented to the American reader in a condensed form, does not still fulfil all the indications which are required by the student of physiology. It is too elaborate, and does not comprehend many new views of importance. The Physiology of Professor Wagner, is both too limited and too abstruse to become familiar with the American student, were it within his reach, and completed. An excellent work on Physiological Anatomy has recently been commenced by Professor Todd and Mr. Bowman, both names ranking high in science; but this is more a work on General Anatomy than Physiology, and moreover is issued in parts, one of which only has appeared in England, leaving the remainder to follow at indefinite periods, after age, perhaps, has somewhat impaired the value of the elder born. The Physiologies of Alison, Mayo, and Elliotson, though containing much that is excellent, especially the former, are in many respects behind the times. In our country we have had several original works on this branch of our science; that of Professor Dunglison ranks deservedly high, and is especially distinguished for its faithful and impartial record of the progress of Physiology.

The scope of the Human Physiology of Dr. Carpenter, is somewhat different from any of the works we have alluded to. It is a clear, compendious resumé of the existing state of Physiological Science, conceived and executed in a genuine philosophical spirit, and peculiarly adapted to the medical student. All the received facts of Physiology are presented in a well-digested form and lucid manner,and the deductions made from them show close reasoning, and a very impartial spirit. The author, though still a young man, has already won a high reputation in this department of medicine. His admirable and original work on General and Comparative Physiology, (see Medical Examiner, vol. ii. p. 233,) has reached a second edition; his numerous contributions to the British and Foreign Review, are generally known and commended; and his recent treatises on Animal and Vegetable Physiology, show varied and profound knowledge, and a discriminating spirit. In a literary point of view, the present work, as well as his other productions, are of a high order; his style is clear, precise and unostentatious, at times rising to positive elegance.

In the present edition numerous additions, and some alterations have been made by the Author and the American Editor. The Physiology of Cells, so important and engrossing a subject at the present moment, is treated of at length in all its relations with nutrition, secretion, and the formation of the tissues.

It is now admitted by every one that the true foundation of Medical Science is an adequate acquaintance with the structure and functions of the human body. The only basis of rational medicine is Physiology. Health is the rule; disease the exception, Pathology must be founded on Physiology; the offices of the different organs must be thoroughly understood, in order that any deviation or derangement may be readily detected; without this knowledge all our endeavours at ascertaining the function in fault will be indefinite and unsuccessful. "The physiologist alone can be the truly able practitioner;" says Dr. Marshall Hall, "That science alone, properly pursued and applied, and not what has been fallaciously, and I fear too often, denominated experience, can lead to a just diagnosis, unfold the nature of disease, suggest new remedies, and guide us in the employment of the old." In physiological medicine, mind and reason, the only true guides in any pursuit or conduct, are both exercised. The so-called system of observation, or the numerical method, which vainly endeavoured to throw these overboard, soon became stranded. This vast abortion, consisting of superstrata piled one above the other into a shapeless and unsightly mass, soon disgusted its original youthful and thoughtless advocates, and has consigned to just oblivion those who stolidly clung to their mishapen offspring.

---

New Preservative for Animal Substances.—A French physician has addressed a paper to the Academy of Sciences on the power of a *syrup of iron* to preserve animal substances unchanged. This syrup is a combination of sugar and iron which does not decompose, crystallise, or ferment, at any temperature. Meats kept in this syrup diminish very little in weight,and resist the most active putrefactive agency.—*London Lancet.*

